# A new large tellinid species of the genus *Pharaonella* from the Ryukyu Archipelago, Japan (Mollusca, Bivalvia)

**DOI:** 10.3897/zookeys.705.12888

**Published:** 2017-10-02

**Authors:** Makoto Kato, Luna Yamamori, Ryutaro Goto, Remi Tsubaki, Ken Ohsuga

**Affiliations:** 1 Graduate School of Human and Environmental Studies, Kyoto University, Yoshida-Nihonmatsu-cho, Sakyo, Kyoto 606-8501, Japan; 2 Seto Marine Biological Laboratory, Field Science Education and Research Center, Kyoto University, 459 Shirahama, Nishimuro, Wakayama 649-2211, Japan; 3 Research and Development Center for Marine Biosciences, Japan Agency for Marine-Earth Science and Technology, 2-15, Natsushima-cho, Yokosuka-city, Kanagawa 237-0061, Japan; 4 1-108 Takano-tamaoka-cho, Sakyo, Kyoto 606-8106, Japan

**Keywords:** Amami Island, bivalve, coral reef ecosystem, sand flat, Tellinidae

## Abstract

A new tellinid species, *Pharaonella amanyu* **sp. n.**, is described from sand banks around Amami Islands, the Ryukyu Archipelago, in southern Japan. A molecular phylogenetic analysis suggests that this new species is closely related to *P. sieboldii*. This species has long siphons and lives buried deep in well-sorted white sand syntopically with *Tonganaella tongana*. These rare, large tellinid species are indicators of unspoiled tidal/subtidal sand flats, which should receive the highest priority conservation in the Ryukyu Archipelago.

## Introduction

Coral reef ecosystems often contain sand flats in which the sediments are mainly composed of coral sand. Even though the biodiversity of tropical coral reefs is both extremely high and the species themselves sometimes endangered (Robert et al. 2002), the biodiversity of the sand flats within coral reef ecosystems has not yet been fully explored. Edateku Island is an uninhabited island facing Amami-Oshima Island across a strait located in a coral reef ecosystem of the northern Ryukyu Archipelago. The east coast of Edateku Island harbours an unspoiled sand flat with well-sorted sand grains inhabited by gobioid sand darts (Tsubaki and Kato 2009). To assess the conservation value of this sand flat, we have conducted an extensive survey of the molluscan biodiversity.

The sand flat is characterised by a diverse fauna of tellinid bivalves, which are surface deposit feeders with colourful thin shells and long extensible siphons. The genus *Pharaonella* (Lamy, 1918) and the recently described *Tonganaella* Huber, Langleit & Kreipl, 2015 are both characterised by large narrow shells with rostration posteriorly, and both occur in tidal and subtidal sandy substrata, where they are buried deeply and extend their long siphons up to the surface of the sediment. In these genera, four species are presently known from Japan: *T. perna* (Spengler, 1798); *T. tongana* (Quoy & Gaimard, 1835); *P. aurea* (Perry, 1811); *P. sieboldii* (Deshayes, 1855). In this survey on Edateku Island, several shells of an unidentified tellinid species belonging to one of these genera were collected. The shells resemble the *Pharaonella* sp. reported from Amami Islands by Nawa (2008) and interpreted as a putative new species. Taxonomic study of this species has been impeded by its rarity and the lack of live specimens for anatomical examination. At last in 2013, a live specimen of this species was found. Morphological and anatomical observations suggest that it belongs to the genus *Pharaonella*. To clarify the specific status and phylogenetic position of the new species, we made molecular phylogenetic analyses of *Pharaonella* species and related taxa in Tellinidae.

In this paper, this bivalve is described as a new species, its special habitat reported, and the conservational priority of the unspoiled intertidal/subtidal sand banks bounding a strait between coral reefs is emphasised.

## Materials and methods

**Figure 1.**
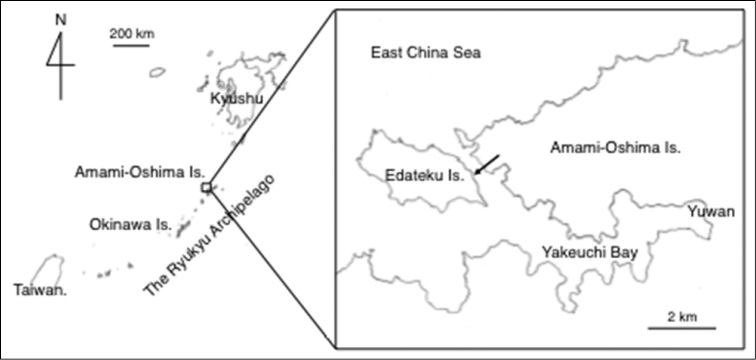
The locality of *Pharaonella amanyu* sp. n. in the Amami Islands, Ryukyu Archipelago. The sand flat constituting the type locality of this new species is shown by an arrow.

**Figure 2.**
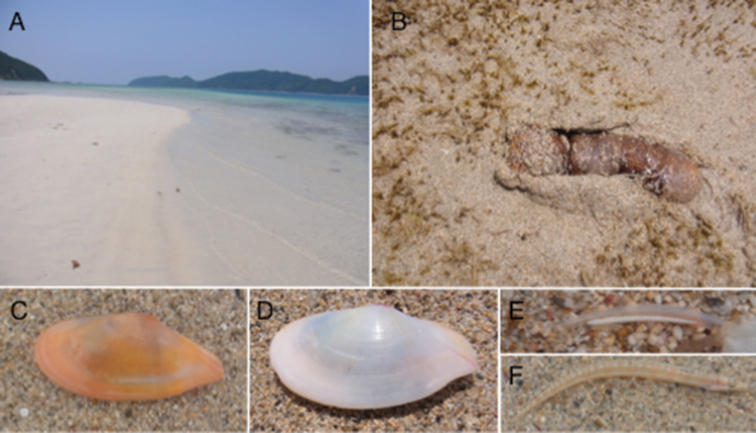
A panorama of the sand flat of Edateku Island at spring low tide (**A**) and the sand-dwelling organisms therein (**B–F**): **B** *Bohadschia bivittata* **C** *Pharaonella amanyu* **D** *Tonganaella tongana* **E** *Branchiostoma belcheri* **F** *Trichonotus elegans*.

An intertidal/subtidal sand flat is present along the eastern coast of Edateku Island in the northern Ryukyu Archipelago, Kagoshima Prefecture, Japan (28°17'26.08"N, 129°13'9.09"E) (Fig. 1). The sediment of the sand flat is well-sorted white sand derived from comminuted corals (Fig. 2A), and the sand flat is inhabited by infaunal organisms, the most characteristic of which are the sea cucumbers *Bohadschia bivittata* (Fig. 2B) and *Holothuria leucospilota* (Holothuriidae), the spatangoid sea urchin *Metalia sternalis* (Brissidae), the lancelet *Branchiostoma belcheri* (Branchiostomidae, Fig. 2E) and the fishes *Kraemeria cunicularia* (Kraemeriidae) and *Trichonotus elegans* (Trichonotidae) (Fig. 2F).

To characterise the molluscan biodiversity of the sand flat, we sampled the molluscs, particularly the tellinid bivalves, by digging the sand with shovels at low tide during the spring tides in May or June each year from 2005 to 2016. Three *Tonganaella*/*Pharaonella* species were found in these samples: *T. perna* (Spengler, 1798), *T. tongana* (Quoy & Gaimard, 1835) (Fig. 2D), plus an unidentified species (Fig. 2C). The third species was very rare, and live specimens were not collected until 26 May 2013. The body of this species was preserved in 99% ethanol, and was utilised for malacological and genetic analyses.

Sequence data were obtained for the mitochondrial cytochrome c oxidase subunit I (COI) gene, nuclear 28S ribosomal RNA (28S rRNA) gene, and nuclear histone 3 (H3) gene of five tellinid species: two *Pharaonella*, two *Tonganaella* and one *Tellinides* species (Table 1). Total DNA was isolated from adductor muscle tissue following a previously described method (Sokolov 2000). We sequenced fragments of the mitochondrial COI gene, 28S rRNA gene and H3 gene. Polymerase chain reaction (PCR) was used to amplify ~700 bp of COI using the universal primers LCO1490/HCO2198 (Folmer et al. 1994), ~1000bp of 28S rRNA gene using the primers D1 (Colgan et al. 2003) and D3 (Vonnemann et al. 2005) and ~350bp of H3 using the primers H3F/H3R (Colgan et al. 1998). Sequencing reactions were performed using the PCR primers for COI gene and H3 gene, and PCR primers and additional sequencing primers, D2F (Vonnemann et al. 2005)/C2R (Dayrat et al. 2001), for 28S rRNA gene, with a BigDye Terminator Cycle Sequencing Ready Reaction Kit (Applied Biosystems, Foster City, CA) on an ABI 3130 sequencer (Applied Biosystems). The obtained sequences were deposited in the DDBJ/EMBL/GenBank databases with accession numbers (Table 1).

**Table 1.** A list of bivalve species analyzed for genetic sequences of three genes (COI, 28S rRNA and H3).

Family	Species	Specimen	GenBank #			Locality
		Catalogue #	COI	28S rRNA	H3	
Tellinidae	*Macoma balthica*	GenBank	KC429141	KC429501	KC429224	
	*Megangulus zyonoensis*	GenBank	JX503037	AB746875	NA	
	*Moerella iridescens*	GenBank	JN398362	AB746876	NA	
	*Pharaonella amanyu* sp. n.	NSMT-Mo 78982 (holotype)	LC311753	LC311747	LC311757	Edateku Is., Kagoshima, Japan
	*Pharaonella sieboldii*	KUZ-Z1880	NA	NA	LC311758	Notojima, Ishikawa, Japan
	*Tellinella crucigera*	GenBank	KC706878	NA	NA	
	*Tellinella cumingii*	KUZ-Z1881	NA	LC311748	LC311759	Edateku Is., Kagoshima, Japan
	*Tellinella virgata*	GenBank/ KUZ-Z1882	AB741079	LC311749	LC311760	Yohena, Okinawa, Japan
	*Scissula similis*	GenBank	KC429142	KC429502	KC429225	
	*Tellinides ovalis*	KUZ-Z1883	LC311754	LC311750	LC311761	Yohena, Okinawa, Japan
	*Tonganaella perna*	KUZ-Z1884	LC311755	LC311751	LC311762	Yohena, Okinawa, Japan
	*Tonganaella tongana*	KUZ-Z1885	LC311756	LC311752	LC311763	Edateku Is., Kagoshima, Japan
Semelidae	*Abra alba*	GenBank	KT307619	KF741656	KC429228	

The sequences were aligned using Muscle (Edgar 2004) as implemented in Seaview software (Galltier et al. 1996; Gouy et al. 2010) using the default settings. Gblocks v0.91b (Castresana 2000; Talavera and Castresana 2007) was employed to eliminate the ambiguously aligned regions of the 28S sequence. The sequence length of the 28S gene before and after Gblocks treatment was 1046 and 1005, respectively. Alignments of the COI gene and H3 gene did not contain insertions or gaps and were therefore unambiguous.

Bayesian and maximum likelihood (ML) phylogenetic analyses were performed based on the combined data set (28S + COI + H3) using MrBayes 3.1.2 (Ronquist and Huelsenbeck 2003) and RAxML version 7.4.2 (Stamatakis 2006) implemented in raxmlGUI ver.1.31 (Silvestro and Michalak 2012). We selected the model GTRGAMMA for RAxML analysis and used the software Kakusan4 (Tanabe 2011) to choose the appropriate models for the MrBayes analysis. The models selected for the MrBayes analysis were GTR_GAMMA for 28S gene, HYK85_GAMMA, GTR_GAMMA and F81_GAMMA for each codon of COI, and GTR_GAMMA for the first codon of H3 gene and J69_Homogenoeous for the second and third codon of H3 gene.

## Systematic account

### Superfamily Tellinoidea Blainville, 1814

#### Family Tellinidae Blainville, 1814

##### *Pharaonella* Lamy, 1918

###### Pharaonella amanyu

Taxon classificationAnimaliaCardiidaTellinidae

Kato & Ohsuga sp. n.

http://zoobank.org/3E9F959B-ED4F-45AC-BE89-6B0F8097039D

**Figure 3.**
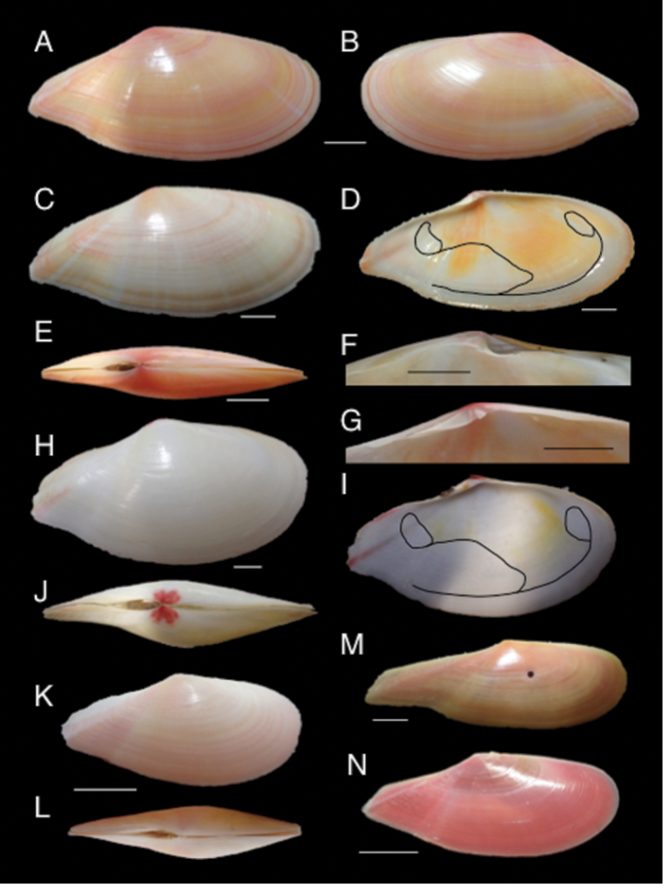
Right (**A, C, F, H, K, M, N**), left (**B, D, G, I**), and paired (**E, J, L**) valves of tellinid species: **A–G** *Pharaonella amanyu* **H–J** *Tonganaella tongana* **K–L** *T. perna* **M** *P. aurea* **N** *P. sieboldii*. Scale bar 10 mm.

**Figure 4.**
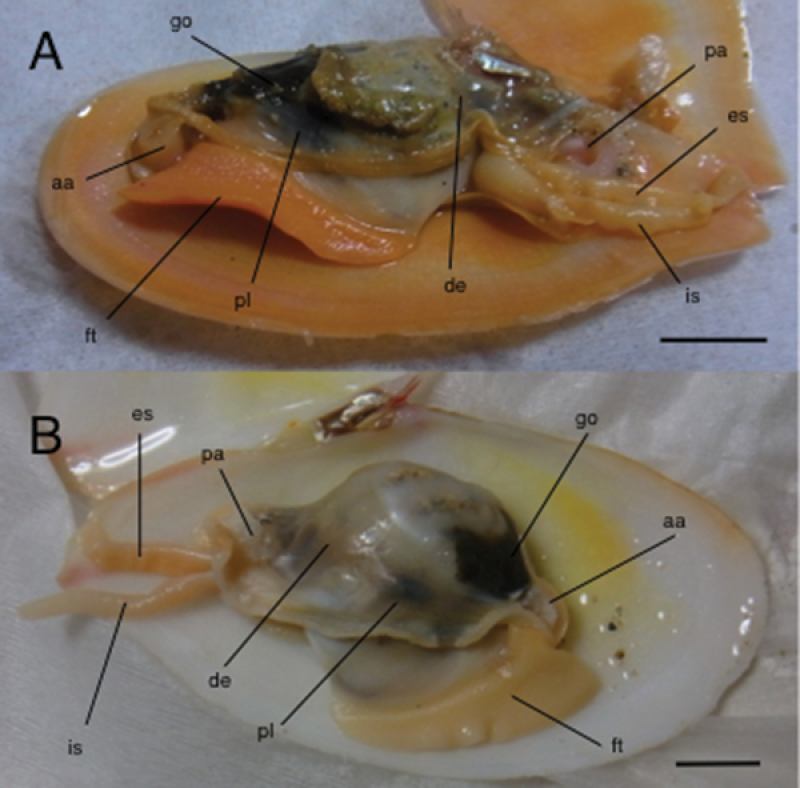
Anatomy of live specimens of *Pharaonella amanyu* (**A**) and *Tonganaella tongana* (**B**). Abbreviations: aa, anterior adductor muscle; de, demibranch; es, excurrent siphon; ft, foot; go, gonad; is, incurrent siphon; pa, posterior adductor muscle; pl, palp. Scale bar 10 mm.

**Figure 5.**
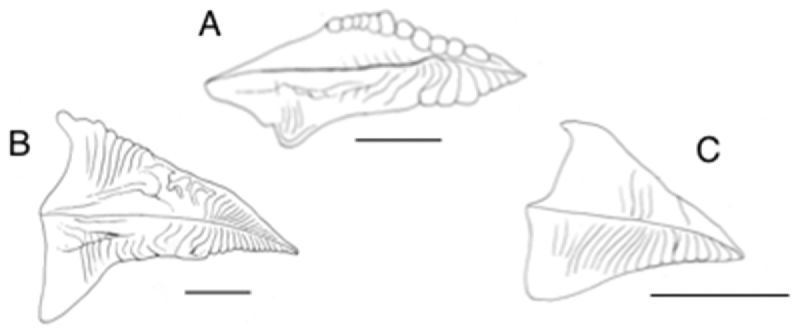
Right hemipalps with outer one reflected: **A** *Pharaonella amanyu* **B** *Tonganaella tongana* **C** *P. sieboldii*. Scale bar 5 mm.

Figs 2C , 3A–G , 4A , 5A

####### Description.

**Shell.** Shell elongate, narrow, subequilateral; inequivalve; anterior section longer than posterior section (Fig. 3A–C); posterior end rostrate, slightly twisted to right (Fig. 3E); weakly gaping posteriorly; left valve glossy, smooth, with faint radials; right valve weakly commarginal ridged on rostrum; exterior color orange to pink, sometimes with fine pale rays emanating from umbo (although the colour of the shell tends to fade once the animal has died); periostracum thin, shiny, slightly iridescent; interior orange to pink with yellow tint in central section; umbones small, posteriorly displaced and touching each other; hinge ligament external, short, and situated in ligamental groove; right valve hinge with 2 elongate lateral teeth, 1 oblique anterior cardinal tooth plus 1 bifid posterior cardinal tooth (Fig. 3F); left valve hinge with 2 distal elongate laterals, 1 trigonal anterior and 1 laminate posterior cardinal tooth (Fig. 3G); posterior edentulous space expanded in both valves; pallial sinus reaching horizontal midline in height of the shell and extending beyond vertical midline in length, confluent with pallial line at middle of shell (Fig. 3D); adductor muscle scars subequal and suborbicular.

**Anatomy.** Mantle and foot orange, thus similar to colour of shell (Fig. 4A), contrasting with creamy white of those of *Tonganaella tongana* (Fig. 4B). Excurrent and incurrent siphons long, similar to each other in length. Siphons able to be extended further than shell length when alive.

Labial palps well developed in comparison with demibranchs. Outer and inner hemipalps elongate-triangular (Fig. 5A), posterior extension of hemipalp weaker than those of *T. tongana* (Fig. 5B) and *P. sieboldii* (Fig. 5C). Inner surfaces of hemipalps have palp folds, which originating from hemipalp intersection spreading toward palp dorsal edge; folds becoming relatively wider distally; distal edges of folds of outer hemipalp form swellings (Fig. 5A).

####### Type material.

**Holotype**: NSMT-Mo 78982, paired valves, length 69 mm, height 32 mm (Figs 3A, B, E, 4A), collected alive by M. Kato on 26 May 2013. **Paratype**: NSMT-Mo 78983, right valve, length 80 mm, height 36 mm (Fig. 3C, G), collected by K. Ohsuga on 28 April 2006; KUZ-Z1878, left valve, length 73 mm, height 33 mm (Fig. 3D, F), same data as the former paratype; KUZ-Z1879, right valve, length 77 mm, height 34 mm, collected by K. Ohsuga at type locality13 July 1995.

####### Type locality.

Edateku Island, Uken, Kagoshima Prefecture, Japan (28°17'26.08"N, 129°13'9.09"E); in sand of subtidal sand bank.

####### Distribution.

In addition to the type locality, the species has also been recorded at Kasari Bay on Amami-Oshima Island, but only by empty shells.

####### Etymology.

The epithet *amanyu* alludes to the mythical archaic peaceful era of Amami Islands, symbolising the undisturbed coastal ecosystem harbouring this bivalve species. It is used as a noun in apposition.

####### Japanese name.

Aman’yu-beni-gai.

####### Habitat.

The bivalve was found deeply buried (about 15 cm) in subtidal sandy bottom.

### Molecular and phylogenetic analyses

**Figure 6.**
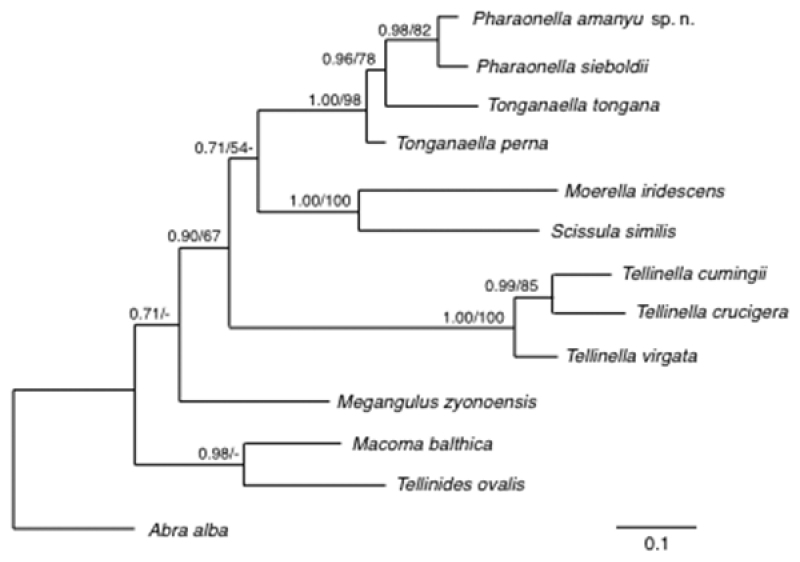
Bayesian tree of *Pharaonella* and *Tonganaella* species based on sequences of COI, 28S rRNA, and H3 genes. The numbers above the branches are Bayesian posterior probabilities followed by maximum likelihood bootstrap support values.

For molecular phylogenetic analysis, COI, 28S rRNA, and H3 sequence data were compared for several *Tonganaella* and *Pharaonella* species (Table 1). To examine the phylogenetic relationships among these species, outgroup species belonging to the Tellinidae and Semelidae were selected from GenBank. The phylogenetic analysis based on the combined data set (COI+28S+H3) (Fig. 6) suggested that *Pharaonella* and two *Tonganaella* species formed a monophyletic clade whereas the *Tonganaella* species were not monophyletic. The tree also suggests that *P. amanyu* is a distinct species most closely related to *P. sieboldii*. The single morphological characteristic that is unique to *Pharaonella* is the set of commarginal grooves on the right shell valve (Huber et al. 2015). In P. amanyu, this sculptural element is present only at the beak.

## Discussion

Both morphological and molecular phylogenetic analyses suggest that the new species should be assigned to the genus *Pharaonella*. The morphological characteristic unique to *Pharaonella* is the commarginally grooved sculpture of right shell valves (Huber et al. 2015), a feature exhibited by the new species. In *P. amanyu* sp. n. this sculpture is confined to the beak of right shell valve. The new species is distinguished from the other Japanese *Pharaonella* species (*P. aurea* and *P. sieboldii*), by their much narrower shells with longer rostrations (Table 2).

Nawa (2008) illustrated a *Pharaonella* sp. specimen collected in Amami Island, and Huber et al. (2015) suggested that this specimen might be *Pharaonella dialeuca* (Deshayes, 1855), a species described from West Malaysia. *Pharaonella dialeuca* differs from *P. amanyu* as follows: (1) the shell of *P. dialeuca* is narrower than *P. amanyu* , (2) *P. dialeuca* has two white rays on shells but *P. amanyu* has numerous thin pale rays, (3) the commarginally grooved sculpture of right shells occurs on the posterior half in *P. dialeuca* but confined in beak in *P. amanyu* (Table 2). In the Philippines, a somewhat similar orange colored tellinid is illustrated as “Tellina (Pharaonella) perna” in Springsteen and Leobrera (1986). The specimen has broad shells (the length/height ratio being 1.94) and so it differs from the narrower *T. perna*, *P. dialeuca*, and *P. amanyu* (Table 2).

Although *P. amanyu* resembles *Tonganaella* species, it can be separated from *T. perna* by the orange colour of shells (white, yellowish or pinkish in *T. perna*) and the long anterior part of the shell (the posterior part is longer than the anterior part in *T. perna*), and from *T. tongana* by the orange colour of the shells and the absence of pink rays at the umbones (Table 2). Our molecular phylogenetic analyses also suggest that the genus *Tonganaella* is not monophyletic and should be synonymised under *Pharaonella*, but this synonymy is not undertaken here because further morphological studies would be necessary.

**Table 2.** Morphological characters of *Tonganaella* and *Pharaonella* species.

Bivalve species	Shell narrowness (shell length/shell height)	Shell shape	Shell color	Sculpture of right valve	Pallial sinus	Distribution
*Pharaonella aurea*	narrowest (2.8–3.0)	anterior part longer than posterior part	bright red or rarely yellow	commarginally ridged	deep	from the Ryukyu Archipelago to Northern Australia
*P. dialeuca*	narrow (2.4)	anterior part longer than posterior part	yellowish orange with two white umbonal divergent rays	commarginally ridged in posterior half	unknown	Philippine Islands
*P. sieboldii*	very narrow (2.3–2.4)	anterior part longer than posterior part	pink	commarginally ridged	deep	around the Japanese Archipelago
*P. amanyu*	narrow (2.1–2.2)	anterior part longer than posterior part	orange with many pale umbonal rays	commarginally ridged only in posterior beak	moderate	around Amami Islands
*Tonganaella perna*	narrow (2.1–2.2)	anterior part shorter than posterior part	white or cream, rarely pink	smooth	moderate	from the Ryukyu Archipelago to Northern Australia
*T. tongana*	least narrow (1.9–2.0)	anterior part as long as posterior part	white, sometimes reddish or yellowish, with pink rays only around unbones	smooth	moderate	from the Ryukyu Archipelago to Northern Australia

It is remarkable that such a large bivalve living near the tidal zone has not been described until now. Among marine bivalves, there are several local endemic species confined to coastal ecosystems in the Ryukyu Archipelago: *Gafrarium yukitai* Habe, 1977 (Veneridae), *Peregrinamor gastrochaenans* Kato & Itani, 2000 (Galeommatidae), and *Merisca monomera* Habe, 1961, *Semelangulus lacrimadugongi* Kato & Ohsuga, 2007 (Tellinidae). The habitats of these endemic species are sandy or muddy tidal flats around estuaries, and the new species may provide another example of these endemic species confined to the Ryukyu Archipelago.

The type locality of the new species is the sand flat at Edateku Island, which is formed by clean white coarse sand lying along a narrow strait and harbours diverse characteristic sand-burrowing organisms such as sea cucumbers, spatangoid sea urchins, lancelets, fishes and molluscs such as terebrid, olivid, and naticid snails, and tellinids. It is noteworthy that one *Pharaonella* and two *Tonganaella* species should be recorded from the sand flat, because many of the tellinid bivalve species are now threatened in the Ryukyu Archipelago (Nawa 2008; Okinawa Prefecture 2017). Although tellinid bivalves are often abundant in seagrass beds within coral reef ecosystems, only small stands of seagrass exist at the Edateku sand flat. In addition to the new species, *Tonganaella tongana* is also a rare species that is now found at only a few sites such as this sand flat and Oura Bay, where reclamation is planned (Diving Team Snack Snufkin 2015). The discovery of the new species, *P. amanyu*, reinforces the significance for conservation value of these sandy habitats in the Ryukyu Archipelago.

Sandy intertidal/subtidal flats and sandbanks form in inland seas along straits where tidal currents are strong (Takasugi et al. 1994). In the Seto Inland Sea of Japan, many sand flats and sand banks were found, harbouring characteristic sand-dwelling organisms such as lancelets (*Branchiostoma belcheri*) and sand lances (*Ammodytes personatus*). Although these sandy habitats are fundamental to ensuring the productivity of coastal waters and to sustaining the fishery resources of inland seas, most such habitats disappeared between 1970–2000 in Japan because of sand mining (Yoshino et al. 2006). In the Amami Islands also, ongoing sand mining is a serious problem for the conservation of coastal ecosystems. To conserve the biodiversity of the sand flat ecosystem of Edateku Island, sand mining must be prohibited in the strait between Edateku Island and Amami-Oshima Islands.

## Supplementary Material

XML Treatment for Pharaonella amanyu
